# Economic evaluation of diagnosis and treatment for latent tuberculosis infection among contacts of pulmonary tuberculosis patients in Thailand

**DOI:** 10.1038/s41598-024-68452-1

**Published:** 2024-07-31

**Authors:** Panida Yoopetch, Olivia Wu, Jiraphun Jittikoon, Montarat Thavorncharoensap, Sitaporn Youngkong, Naiyana Praditsitthikorn, Surakameth Mahasirimongkol, Thunyarat Anothaisintawee, Wanvisa Udomsinprasert, Usa Chaikledkaew

**Affiliations:** 1 Mahidol University Health Technology Assessment (MUHTA) Graduate Program, Mahidol University, Bangkok, Thailand; 2 Health Economics and Health Technology Assessment, School of Health and Wellbeing, University of Glasgow, Glasgow, Scotland, UK; 3 Department of Biochemistry, Faculty of Pharmacy, Mahidol University, Bangkok, Thailand; 4 Social Administrative Pharmacy Division, Department of Pharmacy, Faculty of Pharmacy, Mahidol University, Bangkok, Thailand; 5 Department of Disease Control, Ministry of Public Health, Nonthaburi, Thailand; 6 Information and Communication Technology Center, Office of the Permanent Secretary, Ministry of Public Health, Nonthaburi, Thailand; 7 Department of Clinical Epidemiology and Biostatistics, Faculty of Medicine Ramathibodi Hospital, Mahidol University, Bangkok, Thailand

**Keywords:** Infectious diseases, Medical research

## Abstract

Currently, interferon-gamma release assay (IGRA) is costly and not included as latent tuberculosis infection (LTBI) screening test strategy in Thailand’s Universal Coverage Scheme (UCS) benefit package. The objective of this study was to assess the cost-utility of LTBI screening strategies among tuberculosis (TB) contacts in Thailand. A hybrid decision tree and Markov model was developed to compare the lifetime costs and health outcomes of tuberculin skin test (TST) and IGRA, in comparison to no screening, based on a societal perspective. Health outcomes were the total number of TB cases averted and quality-adjusted life years (QALYs), with results presented as an incremental cost-effectiveness ratio (ICER). One-way and probabilistic sensitivity analyses were performed to explore uncertainties in all parameters. The ICER of TST compared with no screening was 27,645 baht per QALY gained, while that of IGRA compared to TST was 851,030 baht per QALY gained. In a cohort of 1000 TB contacts, both TST and IGRA strategies could avert 282 and 283 TB cases, respectively. At the Thai societal willingness-to-pay threshold of 160,000 baht per QALY gained, TST was deemed cost-effective, whereas IGRA would not be cost-effective, unless the cost of IGRA was reduced to 1,434 baht per test.

## Introduction

Tuberculosis (TB) is a communicable disease caused by *Mycobacterium tuberculosis* bacteria stands as a significant global cause of mortality^1^. Thailand, categorized among the 30 nations grappling with high TB and TB/HIV burdens, recorded an estimated TB incidence of 143 cases per 100,000 population in 2021, resulting in 11 deaths^2^. Approximately 10% of individuals infected with *Mycobacterium tuberculosis* progress to clinically active TB disease, while the remaining 90% enter a latent phase^3^. This latent TB infection (LTBI) represents a substantial reservoir, with a majority progressing to TB disease within the initial 5 years^3^. Globally, LTBI prevalence is 24.8%^4^, with the South-East Asia region reporting the highest at 35%^5^. The World Health Organization's End TB Strategy aims to decrease global TB incidence by addressing both active TB cases and eliminating LTBI through preventive treatment^6,7^. In Thailand, TB preventive treatment is provided for children under 5 years who are living with TB patients. However, the specific number of preventive treatments for household contacts aged 5 years and above remains unreported, and global progress in TB preventive treatment has been modest^8^. Consequently, there is a pressing need to significantly expand access to and provision of TB preventive treatment for individuals aged 5 years and above.

The diagnosis of LTBI relies on identifying an immune response against *Mycobacterium tuberculosis* antigens, achieved through either the tuberculin skin test (TST) or interferon-gamma release assays (IGRA)^9^. TST, a long-standing method particularly prevalent in developing countries, is favored for its cost-effectiveness and minimal infrastructure demands^10^. However, its use in populations with a high prevalence of Bacilli Calmette-Guérin (BCG) vaccination has revealed a drawback in terms of low specificity, leading to false-positive results^11^. TST necessitates a cold chain system and involves two visits for administration and result interpretation^10^. In contrast, IGRA is regarded as more specific than TST, exhibiting no cross-reactivity with BCG vaccination and a lower likelihood of false positives^12^. These tests can be processed in a laboratory, eliminating the need for a second visit. Nevertheless, IGRA is associated with higher costs, technical complexity in execution compared to TST, and requires appropriate laboratory infrastructure^12^.

In Thailand, only TST has been incorporated into the Universal Coverage Scheme (UCS) benefit package, while IGRA are not included due to its higher cost compared to TST. Notably, most economic evaluation studies on LTBI diagnostic tests among TB contacts have been conducted in high-income or upper-middle-income countries. Presently, there has been no economic evaluation of screening strategies for LTBI among contacts aged 5 years and above in Thailand. Therefore, this study aimed to assess the cost-utility of LTBI screening strategies among TB contacts in Thailand. The outcomes of this study can serve as evidence to inform policy decision making on the potential inclusion of IGRA in the UCS benefit package. This information is crucial for supporting strategies aimed at eradicating TB by 2035.

## Methods

### Target population

The model simulated cohorts of individuals aged 5 years and above who were in contact with patients suffering from pulmonary TB. These participants were assumed to be free of active TB and had no underlying comorbidities. The Faculty of Dentistry and Faculty of Pharmacy, Mahidol University, Institutional Review Board (IRB) (MU-DT/PY-IRB 2021/001.1101) committee granted ethics approval for this study and waived the requirement for informed consent. All methods were carried out in accordance with International Guidelines for Human Research Protection such as the Declaration of Helsinki, Belmont Report, CIOMS Guidelines and International Conference on Harmonization in Good Clinical Practice (ICH-GCP).

### Interventions and comparator

The screening strategies, which included TST and IGRA, were evaluated in comparison to a scenario where no systematic screening for LTBI is conducted. For TST alone option, LTBI would be diagnosed using TST. If the TST result is positive, TB preventive treatment would be initiated. For IGRA alone option, this strategy involved the use of IGRA to diagnose LTBI. If the IGRA result is positive, TB preventive treatment would be administered.

### Model structure

A hybrid model, combining a decision tree and a Markov model, was employed to calculate costs and health outcomes of screening strategies during a lifetime horizon with a one-year cycle length. This methodology underwent validation through an expert meeting, and its validity was confirmed by comparing it with models utilized in previously published studies^13^. The decision tree model was adapted from Sohn et al.’s study^14^. The model was designed and run in Microsoft Office Excel 2019 (Microsoft Corp., Redmond, WA).

**Figure 1:**
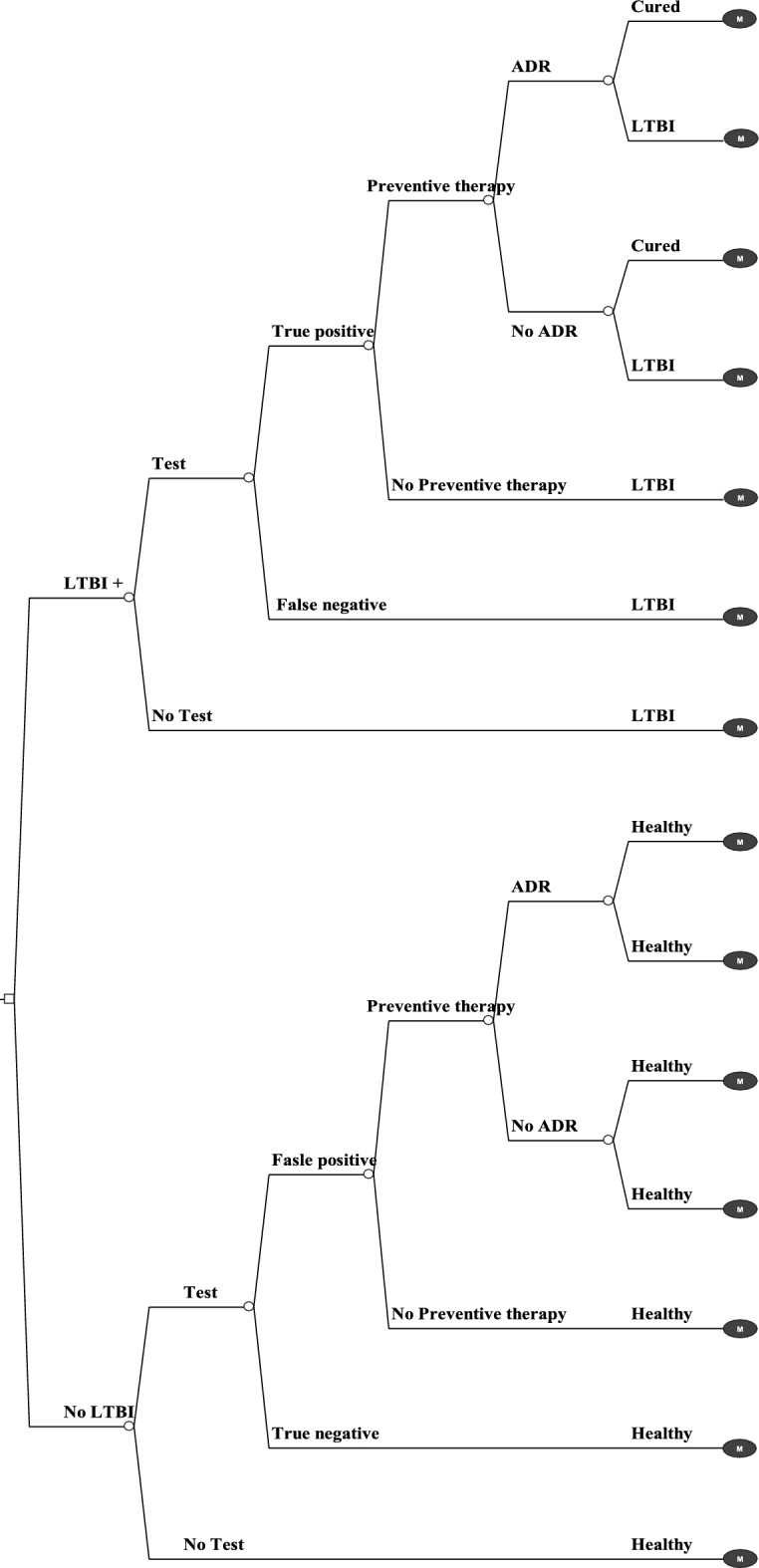
Decision tree model of screening strategy for contacts with pulmonary TB patients.

In the decision tree model, the entry point is from the left, where the entire cohort initiates at time zero as contacts with pulmonary TB patients. Three screening strategies i.e., TST, IGRA and no screening were applied to these contacts. Initially, individuals were categorized into those diagnosed with LTBI and those without LTBI. Individuals might either undergo screening or decline the test either TST or IGRA. For individuals who opted for screening either TST or IGRA, they would enter the diagnostic pathway. They then would progress through the TB preventive treatment, which involved the 3HP (3-month isoniazid and rifapentine) regimen and was stratified by the occurrence of adverse drug reactions (ADR) and cure state. Each decision step in the pathway was accompanied by specific probabilities. This tree structure is replicated for all three screening strategies (Fig. 1). Key assumptions in the decision tree model were as follows. Firstly, all contacts were assumed to be asymptomatic without active TB and HIV infection. Secondly, all individuals with LTBI who progressed to active disease had fully drug-susceptible TB and were treated according to the current clinical guidelines in Thailand. Last, patients who developed ADR i.e., hepatotoxicity completed TB preventive therapy.

**Figure 2:**
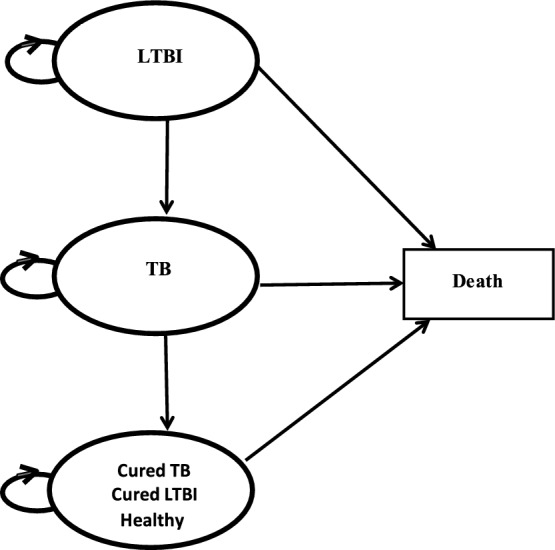
Markov model of individuals with LTBI.

Moreover, our Markov model was adapted from the study of Deuffic-Burban et al.^15^. The model was constructed based on the natural history of individuals with LTBI, encompassing four health states: (1) LTBI, (2) TB, (3) cure/healthy, and (4) death (Fig. 2). The transitional probabilities from one state to another are represented by straight arrows. All contacts entered the Markov model in the LTBI state without receiving any treatments initially. From the LTBI state, contacts could transition to the TB state based on the annual risk of active TB after untreated LTBI. This risk decreased over the first five years after the initial LTBI. Alternatively, individuals in the LTBI state could experience all-cause mortality. In the TB state, contacts could either be cured based on the efficacy of anti-TB drugs or die from TB-related mortality. In the cure/healthy state, individuals could face all-cause mortality. At the end of each cycle, contacts could either remain in the same state, die from all-cause mortality, or progress to the next state. All-cause mortality was applied to all health states (LTBI state, TB state, and cure/healthy state), while only the TB state was associated with TB-related mortality. The model ended when individuals in the cohort died or reached the age of 100 years, aligning with age-specific death rates in the life tables of Thailand^16^. Furthermore, we assumed that individuals with active TB could not revert to LTBI state, and those who had been cured of TB could not transition back to the TB state. It was also assumed that individuals fully adhered to the standard TB treatment regimen. Additionally, the model did not incorporate secondary transmission of TB.

### Model parameters

The model incorporated parameter values for both the decision tree and Markov models, with associated probability distributions required for uncertainty analysis derived from published literatures. Input parameters were categorized into five major groups i.e., epidemiological data, diagnostic test performance, transition probabilities, costs, and utilities (Supplement Table 1).

#### Prevalence and test acceptance

The coverage of contact investigation and the probability of test acceptance was adopted from a study conducted by Imsanguan et al.^17^. In this study, TB patients in Chiang Rai province, Thailand who were invited for screening were interviewed. Prevalence of LTBI were obtained from a meta-analysis of a systematic review conducted by Morrison et al.^18^. Their study included data from 19 studies, and the results indicated that the yield for LTBI in contact investigations was 51.4%^18^.

#### Diagnostic test performance

The performance characteristics of diagnostic tests, specifically sensitivity and specificity, were derived from a systematic review conducted by Pai et al.^19^. The study focused on the results of pooled sensitivity and specificity among BCG-vaccinated participants for QuantiFERON-TB Gold. Additionally, the study utilized results from the pooled sensitivity and specificity among BCG-vaccinated participants for the TST.

#### Treatment efficacy

The completion rates of the 3HP regimen were obtained from a published study conducted by Walker et al.^20^. In this study, patients aged 18 years and above who received the 3HP regimen for LTBI were assessed. The data were collected at Cleveland Clinic, USA, during the period from October 2011 to July 2018. Moreover, the efficacy, specifically in terms of preventing active TB, and the occurrence of ADR (hepatotoxicity) associated with the 3HP regimen were sourced from an updated network meta-analysis conducted by Zenner et al.^21^.

#### Transitional probabilities

The annual risk of active TB following untreated LTBI was obtained from a study conducted by Deuffic-Burban et al.^15^. It was noted that the risk of developing active TB decreased over time, particularly in the first five years after the initial LTBI. The estimated transitional probabilities from the TB state to the Cure state and the TB state to the Death state were obtained from a published literature study conducted by Jittimanee et al.^22^. The data for these transitional probabilities were collected through the Thai National TB program (NTP) over the period from 2001 to 2006.

#### Costs

Considering the societal perspective, direct medical costs and direct non-medical costs were retrieved from published literatures. Nevertheless, indirect costs were excluded to avoid double counting, as morbidity and mortality effects were incorporated into health outcome^24^. The direct medical costs considered in the model included various components such as the costs of diagnostic tests (TST, IGRA), outpatient department (OPD) service for diagnostic tests (TST or IGRA), drug, laboratory tests, inpatient department (IPD) service, directly observed therapy (DOT), TB preventive therapy, and ADR treatment. The cost of TST was obtained from Queen Saovabha Memorial Institute, the Thai Red Cross Society, and was priced at 0.1 ML/dose. The cost of IGRA was obtained from the Ministry of Public Health. The model assumed that TST test required two visits and IGRA test required one visit. The costs associated with OPD visits and chest radiograph were obtained from Namwat et al.^23^.

Regarding the costs of TB preventive therapy, the study utilized a regimen involving 12 once-weekly doses of 3HP. The cost associated with this regimen was obtained from Drug and Medical Supply Information Center, Ministry of Public Health, Thailand^24^. Additionally, the calculation for drug dose was based on the assumption that a patient weighs 40 kg. In addition, the cost of treatment for ADR (hepatotoxicity), was sourced from a retrospective cohort of people with HIV in four Thai hospitals^25^. The model made the assumption that the cost of ADR treatment was specifically linked to hepatotoxicity, indicating that the expenses considered in the economic evaluation were related to managing ADR on the liver^25^. Moreover, the costs of TB treatment included the costs of drugs, laboratory, as well as OPD and IPD services and was sourced from Namwat et al.^23^. The costs of drugs i.e., isoniazid, rifampicin, pyrazinamide, and ethambutol for 2 months, followed by isoniazid and rifampicin for 4 months were calculated for a patient weighing 40 kg. Laboratory cost for TB treatment included six Acid-Fast Bacillus (AFB) and one chest radiographical examination during treatment course. The cost of OPD service represented the monthly follow-up visits through the treatment course at a community hospital. The cost of IPD service was calculated for seven days of hospital stay.

In addition to direct medical costs, the model considered direct non-medical costs for both TB contacts and patients which were obtained from Namwat et al.^23^. For TB patients and contacts, cost of food accounted for providing one meal a day and cost of transportation included expenses related to visits to the hospital during treatment period. Cost of informal care reflected the expenses associated with taking care of TB patients by caregivers. All future costs and health outcomes were adjusted using an annual discount rate of 3%, as recommended by the Thai Health Technology Assessment Guidelines^26^. Costs were converted to Thai baht in the year 2022 by using the Thailand Consumer Price Index (CPI) for medical care^27^.

#### Utilities

Utilities, representing the quality of life for individuals in different health states, were derived from a study conducted by Kittikraisak et al.^28^. In this study, utility scores were obtained from EuroQol 5 Dimensions (EQ-5D) questionnaires administered to a consecutive sample of 222 Thai patients. The utility scores of TB patients who were on TB treatment or completed treatment were 0.69 and 0.88, respectively. However, due to limited data on the utilities of the Thai population with LTBI, we assumed that the utility of the Thai population with LTBI was equal to the utility of TB patients who completed treatment or were cured, which was 0.88^28^.

### Result presentation

Total lifetime costs, total number of people infected with TB (case detected), life years (LYs) and quality-adjusted life years (QALYs) calculated by LYs multiplied by utility score for each screening strategy were presented. The incremental cost-effectiveness ratio (ICER) is defined as the difference in costs divided by the difference in health outcomes either LYs or QALYs between each screening strategy and no screening. If the ICER falls below a Thai societal willingness-to-pay (WTP) threshold, which is set at 160,000 baht per QALY gained, the screening strategy is considered as cost-effective^29^.

### Uncertainty analysis

Both one-way and probabilistic sensitivity analyses (PSA) were conducted to address uncertainties around model parameters. One-way sensitivity analysis involved varying one parameter at a time between its lower and upper limits to test the robustness of the base-case results. The results of this sensitivity analysis were presented graphically in a tornado diagram, illustrating how changes in each parameter individually influenced the model outcomes. Moreover, threshold sensitivity analysis was performed to investigate the cost-effective price of the screening strategy, where the ICER value exceeds the Thai societal WTP threshold of 160,000 baht per QALY gained. Besides, PSA aimed to evaluate the impact of parameter uncertainty on the results by simultaneously stochastically varying all parameters within their respective probability distributions. Monte Carlo simulation was employed, running 1000 iterations to generate a distribution of outcomes. The results of the PSA were presented graphically in a cost-effectiveness acceptability curve, indicating the probability of each strategy being cost-effective across different willingness-to-pay thresholds.

## Results

### Model validation

Model validation involved face validity, with inputs from experts, including four physicians and one nurse specializing in TB treatment as well as a Director of TB Division, Ministry of Public Health, Thailand. These experts assessed and endorsed the conceptual model, input data, and output generation. Additionally, cross-validity testing compared the constructed model with those from a previously published study^13^.

**Figure 3:**
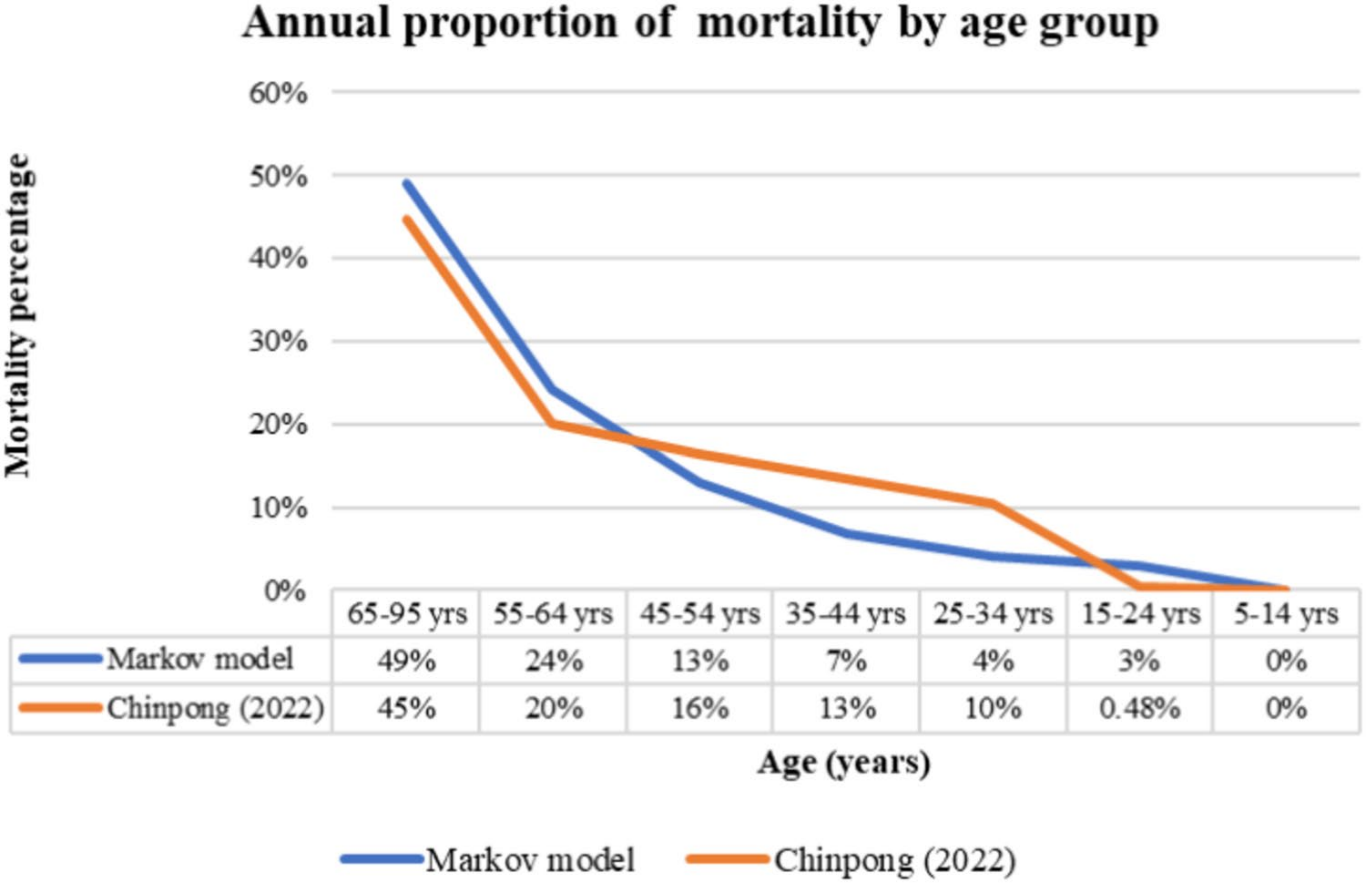
Annual proportion of mortality by age group.

Moreover, to cross validate the model used in our analysis, we analyzed the annual proportion of deaths across different age-groups within the LTBI population. Specifically, we compared the proportions of deaths among individuals aged 25–54 years with those reported in a previous epidemiological study on TB incidence and mortality in Thailand from 2011 to 2022 published by Chinpong et al.^13^, which categorized proportions of deaths into three age groups: 15–24, 55–64, 65 years and older. The study reported mean mortality rates of 0.5% for ages 15–24, 20% for ages 55–64, and 45% for ages 65 and over. We also estimated the mortality rate for individuals aged 25–54 years. As shown in Fig. 3, our findings indicated that the proportions of deaths among individuals aged 25–54 years increased with age, similar to those reported in the previously published study. This consistency supports the validity of our model in reflecting the age-related mortality trends observed in the Thai LTBI population.

### Total costs

Based on a societal perspective, the average total cost for no screening, TST and IGRA strategies were 5,635, 15,860 and 17,296 baht, respectively. The cost of diagnostic tests was the highest in IGRA alone strategy (3,990 baht). The costs of LTBI treatment in TST and IGRA were 11,858 and 11,912 baht, while the cost of anti-TB treatment costs in TST and IGRA were 1,413 and 1,394 baht, respectively.

From a governmental perspective, the average total cost for no screening, TST and IGRA strategies amounted to 3,947, 11,479 and 12,547 baht, respectively. The IGRA alone strategy registered the highest diagnostic test cost at 2,032 baht. The costs of LTBI treatment in TST and IGRA were 9,484 and 9,538 baht, and the expenses for anti-TB treatment in TST and IGRA were 990 and 976 baht, respectively (Table 1).

**Table 1:** Average total cost per person (in 2022 Thai Baht).

Category	Societal perspective			Governmental perspective		
	No screening	TST	IGRA	No screening	TST	IGRA
LTBI diagnosis	0	2,589	3,990	0	1,006	2,032
LTBI treatment	0	11,858	11,912	0	9,484	9,538
Anti-TB treatment	5,635	1,413	1,394	3,947	990	976
Average total cost per person	5,635	15,860	17,296	3,947	11,479	12,547

### Total health outcomes

In a hypothetical cohort of 1000 individuals was assumed to start at age 5 years and older with TB exposure, no screening was projected to result in 514 cases of LTBI, with 376 of these cases progressing to active TB without undergoing treatment for LTBI. TST strategy was anticipated to prevent 282 cases of LTBI and result in the development of 94 cases of active TB. Within this strategy, the breakdown of cases with LTBI treatment, treatment burden, and untreated LTBI amounted to 321, 162, and 193, respectively. Using IGRA was expected to prevent 283 cases of LTBI while leading to the development of 93 active TB cases. Within this approach, the number of cases with LTBI treatment, treatment burden, and untreated LTBI were 325, 16, and 189, respectively. In addition, life years associated with no screening, TST and IGRA strategies were 28.09, 28.47 and 28.48 years, respectively. In terms of QALYs, the values for no screening, TST and IGRA strategies were 24.676, 25.046 and 25.048, respectively.

**Figure 4:**
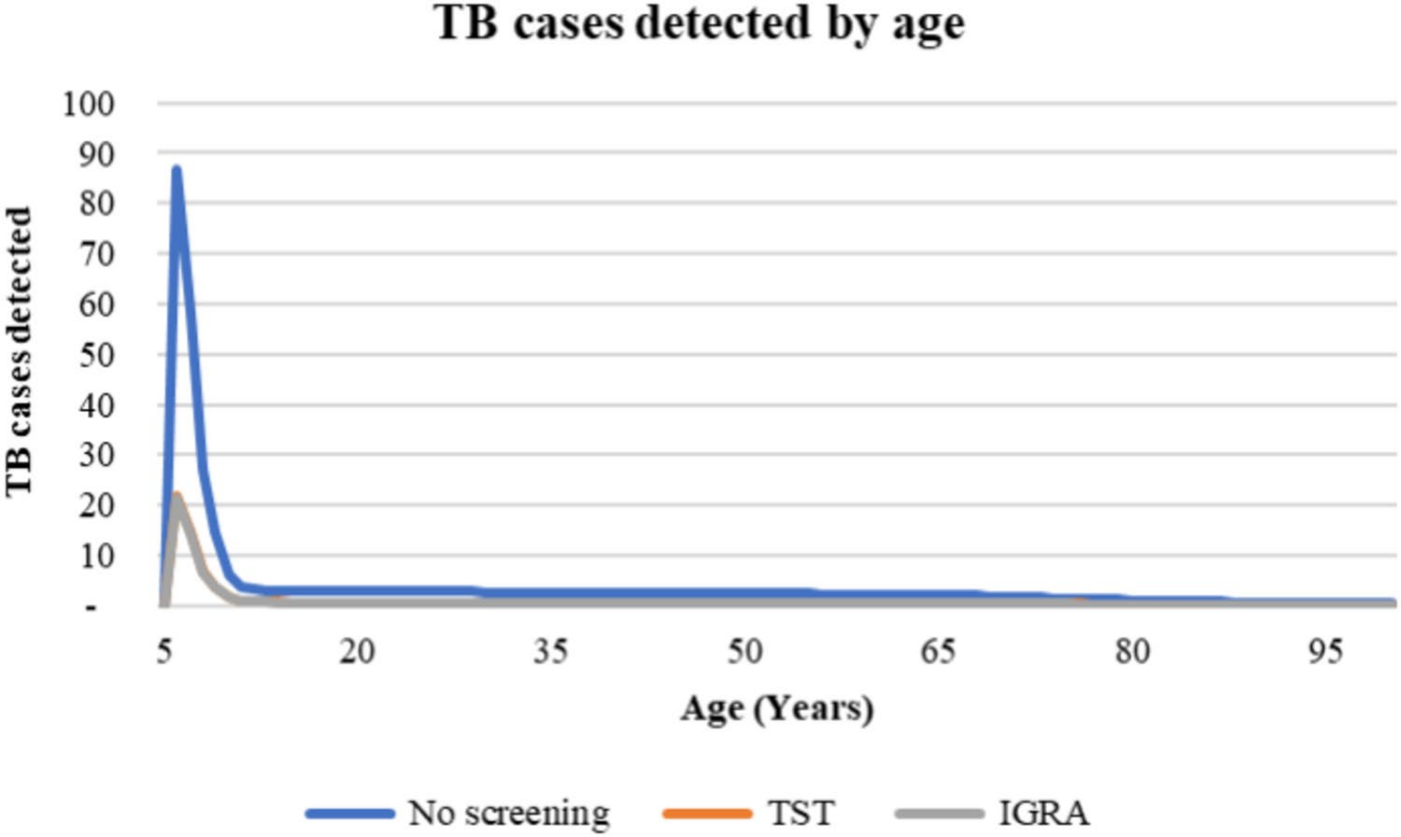
The number of TB cases detected by age.

Figure 4 demonstrates the number of TB cases detected for the three difference strategies (no screening, TST strategy, and IGRA strategy) by age, which was calculated using a Markov model. The model assumed that a cohort of 1,000 individuals with LTBI was followed until the individuals either died or reached the maximum age of 100 years. The model projected the number of TB cases detected over time. No screening was estimated to detect 87, 58, 27, 14 and 6 TB cases over successive five-year intervals. In comparison, the TST strategy was projected to detect 22, 15, 7, 4 and 2 TB cases, while using IGRA was expected to detect 21, 14, 7, 4 and 2 TB cases. Both TST and IGRA strategies detected significantly fewer TB cases compared to no screening, indicating that screening effectively prevents the progression from LTBI to active TB. In addition, the similar numbers of TB cases detected by TST and IGRA suggest that both strategies are comparable in terms of their effectiveness in identifying TB cases among individuals with LTBI.

### Cost-utility analysis

Table 2 presents the cost-utility analysis results. The total costs and QALYs of IGRA strategy were the highest (17,296 baht, 25.048 QALYs) compared to those of TST (15,860 baht 25.046 QALYs) and no screening (5,635 baht, 24.676 QALYs). When compared with no screening, the TST strategy incurred an additional cost of 10,225 baht and provided an additional 0.37 QALYs. The ICER for TST compared to no screening was 27,645 baht per QALY gained, which was below the Thai societal WTP threshold of 160,000 baht per QALY gained, making it cost-effective. In addition, comparing the TST strategy to the next best screening option, IGRA strategy, it was observed that IGRA required an additional cost of 1,436 baht, and yielded a marginal gained of only 0.002 QALYs. The ICER of IGRA compared to TST was 851,030 baht per QALY, which was significantly above the Thai societal WTP threshold, indicating that it was not a cost-effective screening strategy.

**Table 2:** Costs, QALYs and ICER of screening strategies.

Strategy	Total Cost (baht)	Total QALYs	Incremental Cost (Baht)	Incremental QALYs	ICER (baht per QALY gained)
No screening vs. TST					
No screening	5,635	24.676	–	–	–
TST	15,860	25.046	10,225	0.370	27,645
TST vs. IGRA					
TST	15,860	25.046	–	–	–
IGRA	17,296	25.048	1,436	0.002	851,030

### Cos per TB case averted

In a theoretical cohort of 1,000 individuals with TB exposure, the IGRA strategy alone was projected to prevent a greater number of TB cases, totaling 283 cases, compared to TST strategy, which would avert 282 TB cases. However, the IGRA strategy incurred a substantially higher total cost of 17,296 baht, while the TST strategy has a total cost of 15,860 baht. The ICER values for TST and IGRA, relative to no screening, were calculated at 36 and 41 baht per TB case averted, respectively.

### Uncertainty analysis

**Figure 5:**
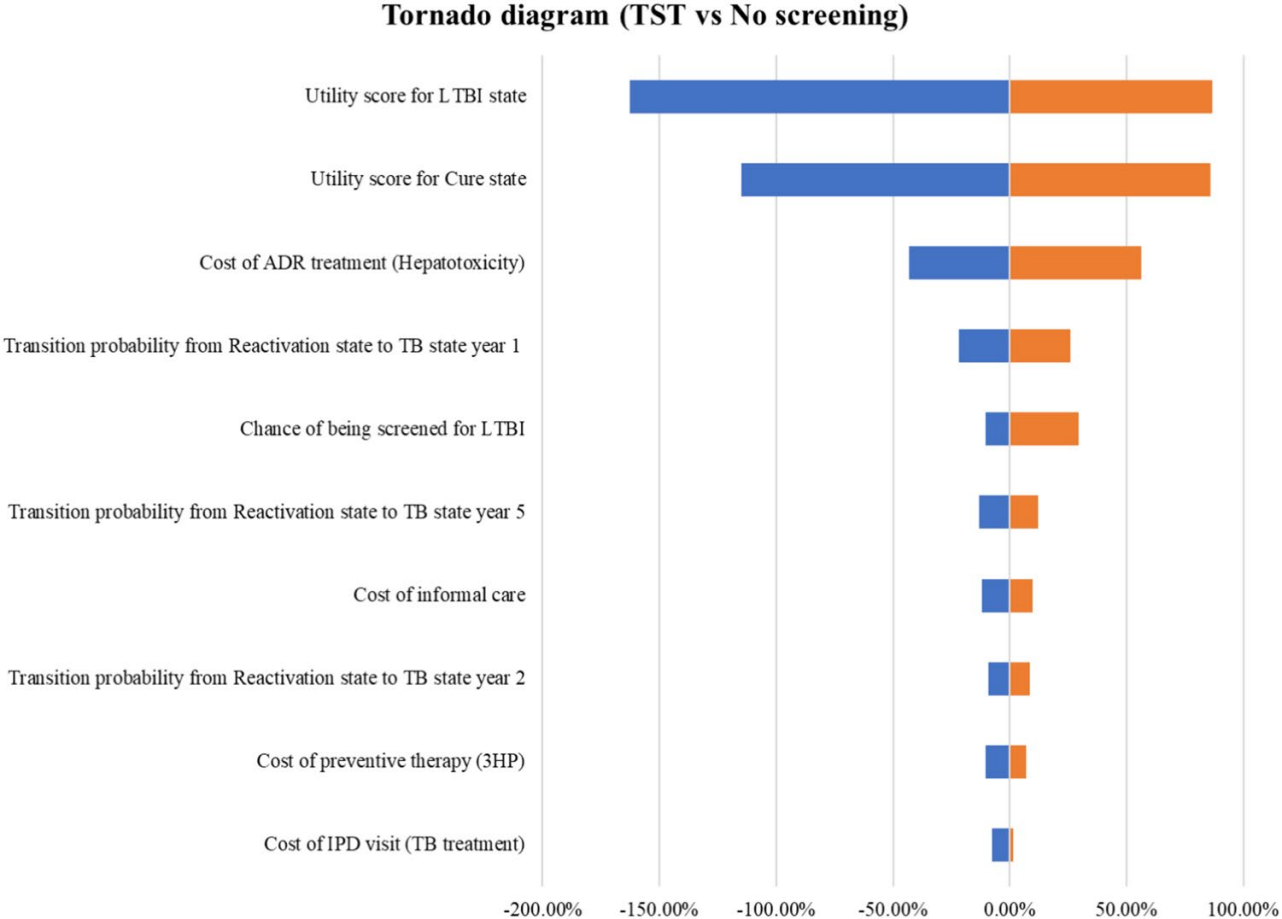
Tornado diagram for TST compared with no screening.

**Figure 6:**
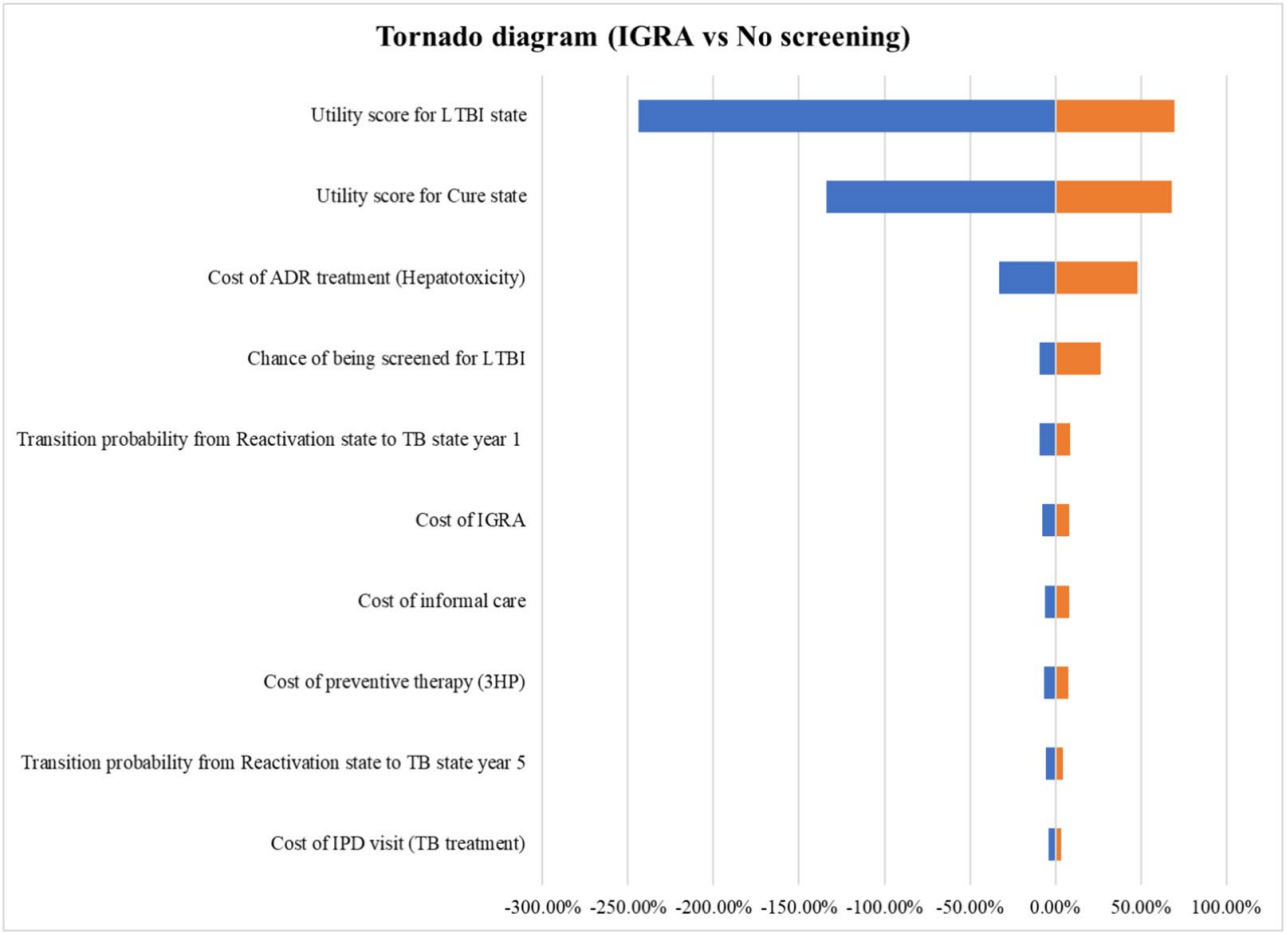
Tornado diagram for IGRA compared with no screening.

Based on the results of one-way sensitivity analysis, the alteration in ICER for both TST and IGRA versus no screening found that the sensitivity of utility score for LTBI state, followed by utility score for cure state and cost of ADR treatment specifically hepatotoxicity, had the most significant effect on the cost-effectiveness of the TST and IGRA compared with no screening in one-way sensitivity analysis. Figure 5 and Fig. 6 show tornado diagrams for TST compared with no screening and for IGRA compared with no screening, respectively. The vertical axis (Y axis) lists the model parameters and the horizontal axis (X-axis) shows the percentage change in the ICER, defined as the difference in costs divided by the difference in health outcomes (QALYs) between TST and no screening. The diagram illustrates the percentage change in the ICER attributable to the change of each individual parameter. In the diagrams, the blue-shaded bars represent a decrease in the ICER value, while the orange-shaded bars represent an increase the ICER value. The ends of the bars indicate the most extreme values used in this analysis.

**Figure 7:**
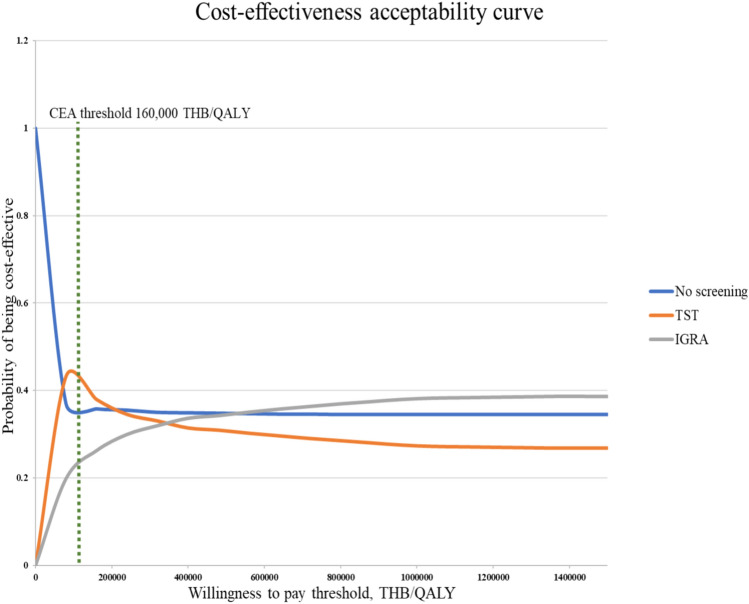
Cost-effectiveness acceptability curve for TST and IGRA compared with no screening.

According to a threshold sensitivity analysis, the cost of IGRA should be reduced from 2,600 to 1,434 baht (45% cost reduction), thus the screening strategy of IGRA would be cost-effective at the WTP 160,000 baht per QALY gained. In addition, cost-effectiveness acceptability curves demonstrated that at a cost-effectiveness threshold of 160,000 baht per QALY gained, the probabilities of TST, IGRA, and no screening being cost-effective were 38%, 26%, and 36%, respectively (Fig. 7).

## Discussion

It is worth noting that the majority of economic evaluation studies on diagnostic tests for LTBI among TB contacts have been conducted in high-income or upper-middle-income countries. This study represents the first attempt to conduct an economic evaluation of screening strategies for LTBI among TB contacts aged 5 years and above in Thailand. Currently, only TST has been incorporated into the UCS’s benefit package, whereas IGRA are excluded due to their higher cost compared to TST. According to our findings, at the Thai societal WTP threshold of 160,000 baht per QALY gained^30^, TST would be more cost-effective compared with no screening, while IGRA would unlikely be cost-effective compared with TST based on a societal perspective.

Our findings align with those of studies conducted in Brazil^31^ and South Korea^14^. In Brazil, a country with a middle-income level and a high burden of TB, the cost-effectiveness of TST, IGRA, and TST followed by IGRA were evaluated from healthcare provider perspective for LTBI screening in TB contacts. Results from a two-year period indicated that TST was identified as the most cost-effective strategy for averting new TB cases^31^. In addition, our results mirror those of a study conducted in South Korea, a high-income country with an intermediate burden of TB. The study evaluated the cost-effectiveness of IGRA, TST and a two-step strategy (IGRA/TST) for individuals testing positive on TST, focusing on high-school adolescents from health system perspective. The findings indicated that TST was the most cost-effective strategy, consistent with our own findings^14^.

In contrast to economic evaluation studies conducted in Switzerland^32^, France^15^, Canada^33^, and Japan^34^, which have low-to-medium TB incidence rates and are classified as high-income countries where the majority of the population receives BCG vaccine, the results have shown that diagnosing LTBI among TB contacts using IGRA was cost-effective. These studies have observed a decrease in total costs with the implementation of IGRA. Despite IGRA being more expensive than TST, its superior specificity has led to a reduction in false positive results. As a result, fewer individuals necessitate treatment for LTBI, and the cost savings associated with IGRA originate from its more accurate targeting of chest X-rays, provision of TB preventive therapy, and management of ADR.

The difference in findings between our study and these studies can be attributed to the higher number of false positive results associated with TST in our study, resulting in 162 cases of unnecessary treatment compared to only 16 cases of unnecessary treatment with IGRA. Despite this, the average total cost of TST for diagnosis and treatment remains relatively low. This discrepancy may be explained by the lower costs associated with TST and the additional preventive TB therapy and management of ADR, which are considerably lower compared to the costs of IGRA. IGRA entails a higher initial cost and requires laboratory infrastructure, resulting in TST costs not being able to compensate for the higher cost of IGRA. Moreover, the cost of the health system in Thailand appears to be relatively lower than in developed countries, which is consistent with the results of the study conducted in Brazil.

Moreover, our study revealed that employing the TST strategy would prevent 282 cases of TB but lead to unnecessary treatment in 162 cases in a hypothetical cohort of 1000 TB contacts. Conversely, utilizing the IGRA strategy would prevent 283 TB cases while resulting in unnecessary treatment in only 16 cases. Despite the current higher cost associated with IGRA for diagnosing LTBI compared to TST, the implementation of IGRA would effectively decrease the number of TB cases and reduce the instances of unnecessary LTBI treatments. However, our findings indicate that at the Thai societal WTP threshold of 160,000 baht per QALY gained, TST was considered cost-effective. In contrast, IGRA would not be deemed cost-effective unless the cost of IGRA was reduced to 1,434 baht per test. Consequently, this could lead to the policy recommendation that at the cost-effective price of IGRA, it should be included in the screening strategy for UCS’s benefit package. This is essential because the currently available LTBI screening strategies in the Thai context are inadequate for TB contacts, particularly when TST is unavailable. Integrating IGRA into the screening strategy would enhance LTBI detection effectiveness and improve the management of TB contacts, thereby contributing to better public health outcomes.

The limitations in this study are needed to be addressed. In this study, we applied Markov model which could not capture TB transmission, meaning that while screening strategies might reduce the incidence of LTBI, they also reduced the number of TB cases and TB transmission. Consequently, the Markov model might underestimate the effectiveness of screening strategies in this study. To mitigate potential biases in this study, systematic reviews, cost data from the Thai context, and model validation were also utilized. Furthermore, the study did not incorporate the costs associated with multidrug-resistant TB, hospitalization due to ADR treatment, and other prevention regimens. Nevertheless, the study conducted sensitivity analyses for TB preventive treatment, ADR treatment, and TB-associated treatment costs, revealing that these parameters had a relatively small impact on the ICER.

## Conclusions

This study suggested that at the Thai societal WTP of 160,000 baht per QALY gained, TST would be more cost-effective compared to no screening. However, IGRA would not be cost-effective compared to TST unless the cost of IGRA is reduced to 1,434 baht, representing a 45% reduction. It is recommended that IGRA, at a cost-effective price, should be incorporated into the UCS’s benefit package as a screening strategy for LTBI among TB contacts aged 5 years and above in Thailand. The results of this study could provide valuable evidence to guide policy decisions regarding the potential inclusion of IGRA into the UCS’s benefit package. This information holds significant importance for supporting strategies aimed at achieving the goal of TB eradication by 2035.

### Supplementary Information

Supplementary Table 1.

## Data Availability

All data generated or analyzed during this study are included in this published article.
