# Supplementary material for: Economic evaluation of diagnosis and treatment for latent tuberculosis infection among contacts of pulmonary tuberculosis patients in Thailand
Source: Sci Rep. 2024 Jul 31;14:17693. doi: 10.1038/s41598-024-68452-1 (PMC11291668; doi:10.1038/s41598-024-68452-1)
Supplement: Supplementary file 1 — Supplementary Table 1. [file 41598_2024_68452_MOESM1_ESM.docx]

**Supplement Table 1** Model parameters

| **Parameter** | **Mean value** | **Standard error** | **Distribution** | **Source** |
| --- | --- | --- | --- | --- |
| **Epidemiologic parameter** |  |  |  |  |
| Chance of being screened for LTBI | 0.81 | 0.162 | Beta | Imsanguan et al. (2020) |
| Prevalence of LTBI | 0.51 | 0.004 | Beta | Morrison et al. |
| **Diagnostic test performance** |  |  |  |  |
| Sensitivity of TST | 0.77 | 0.028 | Beta | Pai et al. (2008) |
| Specificity of TST | 0.59 | 0.054 | Beta | Pai et al. (2008) |
| Sensitivity of IGRA | 0.78 | 0.028 | Beta | Pai et al. (2008) |
| Specificity of IGRA | 0.96 | 0.010 | Beta | Pai et al. (2008) |
| **Treatment efficacy** |  |  |  |  |
| Probability of once-weekly rifapentine and isoniazid (3HP) completion | 0.83 | 0.031 | Beta | Walker et al. (2020) |
| Probability of adverse drug reaction (hepatotoxicity) | 0.008 | 0.001 | Beta | Zenner et al. (2017) |
| Probability of cure | 0.992 | 0.001 | Beta | Zenner et al. (2017) |
| **Transitional probabilities** |  |  |  |  |
| Transition probability from Reactivation state to TB state year 1 | 0.0866 | 0.017 | Beta | Deuffic-Burban et al. (2010) |
| Transition probability from Reactivation state to TB state year 2 | 0.0355 | 0.007 | Beta | Deuffic-Burban et al. (2010) |
| Transition probability from Reactivation state to TB state year 3 | 0.0113 | 0.002 | Beta | Deuffic-Burban et al. (2010) |
| Transition probability from Reactivation state to TB state year 4 | 0.0074 | 0.001 | Beta | Deuffic-Burban et al. (2010) |
| Transition probability from Reactivation state to TB state year 5 | 0.0024 | 0.000 | Beta | Deuffic-Burban et al. (2010) |
| Transition probability from TB state to Cure state | 0.63 | 0.003 | Beta | Jittimanee et al. (2009) |
| Transition probability from TB state to Death state | 0.08 | 0.002 | Beta | Jittimanee et al. (2009) |
| **Costs** |  |  |  |  |
| ***Direct medical costs*** |  |  |  |  |
| Cost of TST | 280 | 56 | Gamma | Queen Saovabha Memorial Institute (2022) |
| Cost of OPD visit for TST | 290 | 58 | Gamma | Namwat et al. (2017) |
| Cost of IGRA | 2600 | 520 | Gamma | MOPH (2022) |
| Cost of OPD visit for IGRA | 163 | 33 | Gamma | Namwat et al. (2017) |
| Cost of Chest radiograph | 290 | 58 | Gamma | Namwat et al. (2017) |
| Cost of OPD visit (chest radiograph) | 145 | 29 | Gamma | Namwat et al. (2017) |
| Cost of preventive therapy (3HP) | 2352 | 470 | Gamma | Drug and Medical Supply Information Center, MOPH (2022) |
| Cost of ADR treatment (Hepatotoxicity) | 6697 | 2612 | Gamma | Maleewong (2008) |
| Cost of Drug (TB treatment) | 931 | 186 | Gamma | Namwat et al. (2017) |
| Cost of Lab (TB treatment) | 813 | 163 | Gamma | Namwat et al. (2017) |
| Cost of OPD visit (TB treatment) | 1480 | 296 | Gamma | Namwat et al. (2017) |
| Cost of IPD visit (TB treatment) | 6155 | 1231 | Gamma | Namwat et al. (2017) |
| Cost of DOT (TB treatment) | 2385 | 477 | Gamma | Namwat et al. (2017) |
| Cost of Home visit (TB treatment) | 4016 | 803 | Gamma | Namwat et al. (2017) |
| ***Direct non-medical costs*** |  |  |  |  |
| Cost of Food | 112 | 22 | Gamma | Namwat et al. (2017) |
| Cost of travel for follow up | 115 | 23 | Gamma | Namwat et al. (2017) |
| Cost of informal care | 565 | 113 | Gamma | Namwat et al. (2017) |
| Cost of Food (TB treatment) | 674 | 135 | Gamma | Namwat et al. (2017) |
| Cost of travel for follow up  (TB treatment) | 689 | 138 | Gamma | Namwat et al. (2017) |
| Cost of informal care (TB treatment) | 3386 | 677 | Gamma | Namwat et al. (2017) |
| Cost of travel for DOT (TB treatment) | 1999 | 400 | Gamma | Namwat et al. (2017) |
| **Utility** |  |  |  |  |
| Utility score for LTBI state | 0.88 | 0.176 | Beta | Kittikraisak et al. (2012) |
| Utility score for TB state | 0.69 | 0.138 | Beta | Kittikraisak et al. (2012) |
| Utility score for Cure state | 0.88 | 0.176 | Beta | Kittikraisak et al. (2012) |
